# TGFβ Signaling in the Pancreatic Tumor Microenvironment

**DOI:** 10.3390/cancers13205086

**Published:** 2021-10-11

**Authors:** Daniel R. Principe, Kaytlin E. Timbers, Luke G. Atia, Regina M. Koch, Ajay Rana

**Affiliations:** 1 Medical Scientist Training Program, University of Illinois College of Medicine, Chicago, IL 60612, USA; 2 Department of Surgery, University of Illinois at Chicago, Chicago, IL 60607, USA; ktimbers@umich.edu (K.E.T.); lukeatia@gmail.com (L.G.A.); rkoch3@uic.edu (R.M.K.); 3 Jesse Brown Veterans Affairs Hospital, Chicago, IL 60612, USA

**Keywords:** pancreatic cancer, TGFβ signaling, tumor microenvironment

## Abstract

**Simple Summary:**

There is currently no effective treatment for patients with advanced pancreatic ductal adenocarcinoma (PDAC). Transforming Growth Factor β (TGFβ) signaling has been implicated in several hallmark features of PDAC pathobiology, and TGFβ inhibitors are beginning to show promise in the treatment of PDAC. Here, we discuss the known roles of TGFβ signaling in the pancreatic tumor microenvironment, as well as clinical trials evaluating TGFβ pathway inhibitors in PDAC patients.

**Abstract:**

Pancreatic ductal adenocarcinoma (PDAC) is associated with poor clinical outcomes, largely attributed to incomplete responses to standard therapeutic approaches. Recently, selective inhibitors of the Transforming Growth Factor β (TGFβ) signaling pathway have shown early promise in the treatment of PDAC, particularly as a means of augmenting responses to chemo- and immunotherapies. However, TGFβ is a potent and pleiotropic cytokine with several seemingly paradoxical roles within the pancreatic tumor microenvironment (TME). Although TGFβ signaling can have potent tumor-suppressive effects in epithelial cells, TGFβ signaling also accelerates pancreatic tumorigenesis by enhancing epithelial-to-mesenchymal transition (EMT), fibrosis, and the evasion of the cytotoxic immune surveillance program. Here, we discuss the known roles of TGFβ signaling in pancreatic carcinogenesis, the biologic consequences of the genetic inactivation of select components of the TGFβ pathway, as well as past and present attempts to advance TGFβ inhibitors in the treatment of PDAC patients.

## 1. Introduction

Despite significant progress for several difficult-to-treat malignancies in recent years, there is currently no effective treatment for pancreatic ductal adenocarcinoma (PDAC). While broad-spectrum chemotherapy can modestly extend survival for most patients, nearly all will eventually progress during treatment [1], and overall five-year survival remains at a dismal 10% [2]. The poor clinical outcomes associated with PDAC largely stem from a late stage of diagnosis, the lack of an effective screening modality, and widespread drug resistance [1,3,4]. This highlights an urgent need for new therapeutic approaches in order to improve outcomes for what is largely considered an incurable disease. To this end, transforming growth factor β (TGFβ) is emerging as a key mediator of the PDAC tumor microenvironment (TME).

In benign and neoplastic pancreatic epithelial cells, TGFβ signaling leads to cell cycle arrest [5,6], predominantly via the transcriptional upregulation of cyclin-dependent kinase inhibitors [7]. In advanced PDAC tumors, this function is often lost [8], and levels of TGFβ begin to positively correlate with recurrence and negatively correlate with disease-free survival [9]. TGFβ is a potent and pleiotropic cytokine with several context-specific and often contradictory functions in PDAC, altering cell proliferation, differentiation, and motility, as well as processes within the tumor microenvironment such as fibrosis and immune evasion. Here, we discuss the known elements of TGFβ signaling in PDAC, as well as past and present attempts to advance therapies targeting the TGFβ pathway in clinical trial.

## 2. Canonical TGFβ Signaling in PDAC

In the canonical TGFβ signaling pathway, TGFβ family ligands associate with the N-terminal extra-cellular ligand-binding ectodomain of the type 2 TGFβ receptor (TGFBR2). TGFBR2 then recruits the type 1 TGFβ receptor (TGFBR1), which phosphorylates SMAD2 and SMAD3 proteins via its serine/threonine kinase domains [5,10,11]. Following phosphorylation by TGFBR1, SMAD2, and SMAD3 form a heteroligomer with SMAD4, which then traffics to the nucleus [5,10,11]. Here, SMAD4 will associate with CAGAC motifs (or SMAD-binding elements) via its MH1 domain and alter gene expression in a context-specific manner [12,13]. In benign and neoplastic pancreatic epithelial cells, the TGFβ/SMAD pathway arrests the cell cycle mainly via the transcriptional upregulation of cyclin-dependent kinase (CDK) inhibitors, blocking the transition from G1 to S phase by repressing cyclin–CDK complexes [14].

SMAD-mediated TGFβ signaling has been extensively explored in pancreatic epithelial cells, and is generally considered, at least in part, tumor suppressive. Importantly, mice with the pancreas-specific ablation of either TGFBR2 or SMAD4 show enhanced tumor formation and early mortality [15,16,17]. Furthermore, TGFβ signaling has been shown to impede the anchorage-independent growth of SMAD4-expressing CaPan1 cells via the upregulation of the CDK inhibitor p15^INK4b^ (p15), though this was not observed in CFPac-1 cells with the functional inactivation of SMAD4 [18,19,20]. Similar results were observed in vivo, as the loss of *Smad4* cooperated with oncogenic alterations to *Kras*, *Trp53*, or *Cdkn2a* to induce PDAC in adult mice, largely due to the deregulation of the *Cdkn2b* gene product p15 [21].

Growth-suppressive TGFβ signaling also involves the upregulation of the CDK inhibitor p21^CIP1/WAF1^ (p21), which was not observed in PDAC cell lines lacking either SMAD4 or TGFBR2 [14,22]. In non-malignant pancreatic epithelial cells, p21 is an established tumor suppressor gene required for TGFβ-induced cell cycle arrest [23] and that opposes acinar-to-ductal metaplasia and early pancreatic carcinogenesis in vivo [24]. This is consistent with clinical observations that p21 positively associates with both SMAD4 and TGFβ1 [25], and that PDAC patients with robust p21 expression have improved prognosis [25].

To this end, *SMAD4* is among the most frequently altered genes in PDAC. The *SMAD4* gene (also known as *deleted in pancreatic cancer locus 4* or *DPC4*) is located on chromosome 18q21, and is lost in roughly 55% of pancreatic cancers mainly via homozygous deletion or by intragenic mutations and the subsequent loss of heterozygosity [26]. The loss of *SMAD4* leads to extensive signaling alterations in PDAC cells, including the disruption of TGFβ-induced cell cycle arrest, enhanced tumor cell migration, and reduced chemo- and radiosensitivity [22,27,28,29,30,31]. Accordingly, SMAD4 status is an established independent prognostic biomarker in PDAC. For example, in patients undergoing pancreaticoduodenectomy, SMAD4 expression predicted improved survival even when adjusted to other prognostic factors including stage, tumor size, surgical margins, lymph node status, and the use of adjuvant chemoradiotherapy [32]. Similar results have been observed in subsequent studies, many of which suggest that the loss or functional inactivation of SMAD4 is associated with poor survival, increased lymph node involvement and/or distant metastases, and higher rates of treatment failure [33,34,35,36,37,38,39].

Though the clinical association between SMAD4 and poor prognosis is now supported by several meta-analyses [39,40,41,42], the mechanisms through which the loss of SMAD4 signals enhance PDAC tumorigenesis are still emerging. Beyond its effects on cell cycle regulation, the TGFβ/SMAD pathway is a recognized mediator of epithelial-to-mesenchymal transition (EMT), tumor cell migration, and metastasis [5]. In human pancreatic ductal epithelial cells, SMAD4 is necessary for TGFβ-induced N-cadherin expression, and SMAD4-knockdown impairs TGFβ-induced migration and invasion [43]. Although much of TGFβ-induced migration appears to involve non-SMAD signals (discussed in detail below), the SMAD pathway appears to have important roles in the pro-metastatic aspects of TGFβ signaling [44].

In vitro studies demonstrate that the complete loss of SMAD4 enhances the malignant transformation of human pancreatic ductal epithelial cells and enhanced metastasis in orthotopic xenograft experiments. The subsequent restoration of SMAD4 re-established the sensitivity of these cells to the growth inhibitory effects of TGFβ, increased tumor latency, and decreased metastasis [45]. In PDAC specimens, SMAD3 upregulation was associated with several features of aggressive disease including a higher tumor grade, lymph node metastasis, increased EMT-like features, and shorter survival [46]. Consistently with these observations, SMAD3 knockdown increased E-cadherin expression, downregulated Vimentin, and reduced cell migration cells in vitro, as well as prevented TGFβ-induced EMT in SMAD4-expressing tumor cells [46]. However, SMAD control over EMT is complex and often contradictory, as cell lines harboring SMAD4^Y353C^, a missense mutation affecting the MH2 domain, had enhanced cell migration and invasion without increased proliferation in vitro. Additionally, cells with the SMAD4^Y353C^ mutation demonstrated decreased E-cadherin and increased Vimentin expression compared to those with overexpression of wild-type SMAD4 [47].

SMAD signaling appears to induce EMT largely via the transcriptional regulation of select target genes. For example, SMAD4 can directly upregulate the EMT drivers ZEB1 and SNAIL and inhibit the expression of E-Cadherin [48]. Additionally, during EMT, SMAD3 and SMAD4 can form a transcriptional repressor complex with Snail1, also inhibiting the expression of E-Cadherin as well as the tight junction protein CAR [49]. Recently, SMAD4 has been identified as a transcriptional repressor of FOSL1 in PDAC. Tumor cells with a loss of SMAD4 displayed increased FOSL1 expression which is both necessary and sufficient to enhance the metastatic colonization of the lungs [50]. However, while generally considered a pro-tumorigenic effect and an obligate step to metastasis, TGFβ-induced EMT has also been linked to the more tumor-suppressive elements of TGFβ signaling. In TGFβ-sensitive PDAC cells, EMT can be lethal by converting TGFβ-induced SOX4 from being tumor-permissive to pro-apoptotic. While SMAD4 was required for TGFβ-induced EMT, TGFβ-induced SOX4 expression occurred independent of the SMADs. The authors therefore concluded that tumor suppressive TGFβ signaling functions through the EMT-mediated disruption of a lineage-specific transcriptional network [51]. Recent evidence also appears to implicate Prostate Apoptosis Response-4 (Par-4) in this process [52], though other potential mediators are largely unknown.

Beyond EMT and metastasis, TGFβ/SMAD signaling also interacts with several other signaling networks and cell processes. For example, endogenous TGFβ signaling is considered oncogenic in tumor cells lacking SMAD4 [53]. Accordingly, PDAC cells with a loss of SMAD4 display an increased expression of vascular endothelial growth factor (VEGF), epidermal growth factor receptor (EGFR), and the stemness marker CD133, as well as the hyper-activation of the ERK, p38, and AKT pathways [29]. Additionally, SMAD4 has been proposed as a barrier to ERK and WNT-driven oncogenesis [54]. Notably, SMAD4 interacts with the metabolic enzyme and WNT target Glycogen Synthase Kinase 3 (GSK3), which preferentially phosphorylates select SMAD4 mutants to reversibly inhibit TGFβ/SMAD signaling [55]. This is largely consistent with previous reports suggesting that GSK3 integrates FGF, WNT, and TGFβ signaling pathways [56]. In addition to crosstalk with GSK3, SMAD4 also interacts with the glycolytic enzyme Phosphoglycerate Kinase 1 (PGK1) in PDAC. The loss of SMAD4 therefore leads to PGK1 overexpression, enhancing both glycolysis as well as oxidative phosphorylation to accelerate tumorigenesis [57]. Accordingly, the loss of SMAD4 leads to extensive metabolic reprogramming in PDAC cells, which appears to alter tumor cell sensitivity to mitochondrial-targeted therapy [58].

Finally, SMAD4 is also a central regulator of autophagy in PDAC [59]. Tumor cells with a loss of SMAD4 display increased levels of reactive oxygen species (ROS) and radiation-induced autophagy, thereby limiting the tumoricidal effects of radiation in vivo [31]. Given the importance of autophagy in several aspects of pancreatic tumorigenesis [60], this may provide the opportunity for therapeutic intervention for PDAC patients with SMAD4 loss, particularly in light of a recent retrospective study suggesting that the addition of the autophagy inhibitor hydroxychloroquine may improve treatment responses in SMAD4-null PDAC [61]. Hence, the intersection between SMAD4 and autophagy warrants continued exploration, particularly in light of new evidence supporting the combination of hydroxychloroquine and ERK pathway inhibition in PDAC [62].

Finally, recent evidence also appears to support a reciprocal interplay between the SMAD pathway and circadian rhythms. In SMAD4-expressing PDAC cells, mRNA transcripts for *TGFβ1*, *SMAD3*, *SMAD4*, and *SMAD7* oscillate in a circadian fashion, which is impaired by altering genes involved in regulating the circadian rhythm. The SMAD pathway also exerted transcriptional control over the clock genes *DEC1*, *DEC2*, and *CRY1*, and activation of the canonical TGFβ pathway resulted in an altered clock accompanied by cell cycle arrest, increased apoptosis as well as evasion, and enhanced sensitivity to gemcitabine [63].

## 3. Non-Canonical TGFβ Signaling in PDAC

**Figure 1:**
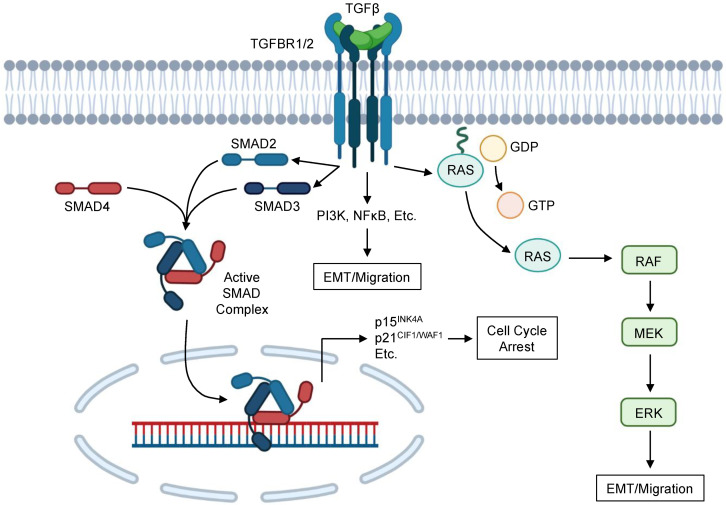
Simplified schema of TGFβ signaling in PDAC cells. TGFβ family ligands associate with the N-terminal extra-cellular ligand-binding ectodomain of the type 2 TGFβ receptor (TGFBR2), which then recruits the type 1 TGFβ receptor (TGFBR1). TGFBR1 phosphorylates SMAD2 and SMAD3 proteins via its serine/threonine kinase domains, leading to the formation of a heteroligomer with SMAD4. This activated SMAD complex then translocates to the nucleus, and SMAD4 associates with CAGAC motifs (or SMAD-binding elements) via its MH1 domain to alter gene expression. In benign and neoplastic pancreatic epithelial cells, the TGFβ/SMAD pathway can arrest the cell cycle via transcriptional upregulation of cyclin-dependent kinase (CDK) inhibitors including p15^INK4A^, p21^CIF1/WAF1^, and others. This blocks the transition from G1 to S phase by repressing cyclin–CDK complexes, as well as direct several additional cellular processes as described in this review. TGFβ signaling also involves several non-SMAD components, most notably the RAS/RAF/MEK/ERK pathway, which occurs through the direct phosphorylation of ShcA. TGFβ signaling also involves crosstalk with several additional pathways, including PI3K, NFκB, and many others. Although the biologic effects of these signaling events are varied, most non-SMAD TGFβ signaling appears to facilitate epithelial-to-mesenchymal transition (EMT) and cell migration.

In addition to the well-studied SMAD pathway, TGFβ signaling involves several non-SMAD signaling elements (Figure 1). This is particularly true for pro-EMT and pro-migratory TGFβ signaling, which is mediated by both SMAD-dependent and SMAD-independent mechanisms [49,64,65]. Non-SMAD TGFβ signaling is highly complex and appears to involve crosstalk with several other signaling cascades. For instance, TGFβ signaling activates the ERK/MAPK pathway through the direct phosphorylation of ShcA [66]. In the pancreas, mice lacking either TGFBR1 or TGFBR2 show diminished ERK activation, even in the presence of an oncogenic KRAS mutation, suggesting that TGFβ signals are required for ERK activation in the pancreas [67]. ERK signaling is generally considered oncogenic, accelerating tumor formation by enhancing proliferation, EMT, migration, and invasion [68]. In non-small cell lung cancer (NSCLC) cells, the pharmacologic inhibition of ERK activation led to more epithelial phenotypes, prevented TGFβ-induced EMT, and increased sensitivity to EGFR inhibition [69]. Similar results have been observed in mammary gland, cortical tubule [70], renal tubule [71], and colon cancer epithelial cells [72]. Accordingly, ERK is required for TGFβ-induced EMT in non-malignant pancreatic ductal epithelial cells, early neoplastic epithelial cells, and SMAD4-expressing tumor cells [67].

However, despite the known tumor-permissive effects of ERK signals, ERK has also been implicated in several additional cell processes including senescence, autophagy, and apoptosis [73]. Though classically mitogenic, ERK signaling can also induce p21 expression in tumor cells, thereby leading to cell cycle arrest [74]. Additionally, RAS activation can stabilize p21 by promoting the formation of p21/cyclin complexes and preventing proteasomal degradation [75]. In non-malignant pancreatic ductal epithelial cells, ERK is required for the TGFβ-induced expression of p21, and the pharmacologic inhibition of ERK activation prevents the formation of TGFβ-induced complexes between p21 and CDK2 [67]. ERK was not required for TGFβ-induced p21 expression in cells harboring an oncogenic KRAS^G12D^ mutation, although ERK still facilitated the formation of p21/CDK2 complexes [67]. However, in SMAD4-expressing PDAC cells, ERK activation had no effect on TGFβ-induced p21 expression. Additionally, though TGFβ enhanced p21 expression, TGFβ failed to promote complexes between p21 and CDK2 unless cells also underwent the pharmacologic inhibition of ERK activation [67].

In addition to acting downstream of TGFβ, ERK activation can also alter TGFβ sensitivity in PDAC cells, namely through the regulation of KLF11. KLF11 functions to repress TGFβ-induced transcription of SMAD7 by recruiting mSin3a via GC-rich sites at the promoter region [76]. As SMAD7 is an inhibitory SMAD protein that impedes the transmission of canonical TGFβ signaling through a negative feedback loop [77], KLF11 is considered a negative regulator of the TGFβ pathway [76]. In PDAC cells, ERK activation leads to the downregulation of KLF11, thereby potentiating the effects of TGFβ through the termination of the negative feedback loop imposed by SMAD7 [76]. Interestingly, crosstalk between TGFβ and ERK signals also have been reported to feed into additional signaling networks, including the PI3K/AKT pathway. In PDAC cells with a loss of SMAD4, incubation with TGFβ led to the downregulation of PTEN, which was ablated upon MEK1 inhibition [78]. However, ERK also appears to antagonize the TGFβ-induced upregulation of the tumor suppressor gene *Lefty* [79], again suggesting that the interactions between TGFβ and ERK signals are both complex and highly context-dependent [79].

Beyond the ERK pathway, TGFβ signaling in PDAC involves crosstalk with several other signaling networks and cellular processes, many of which have been studied in the setting of TGFβ-induced EMT. For example, several recent studies have identified alterations in the ROS pathway associated with TGFβ-induced EMT. TGFβ alters tumor mitochondrial function during EMT, increasing both total mitochondrial mass and ROS production [80]. This is consistent with additional evidence suggesting that NOX4-derived ROS signaling contributes to TGFβ-induced EMT in pancreatic cancer cells through the redox sensor PTP1B [81], as well as the observation that TGFβ cooperates with the redox protein Nrf2 to promote EMT in pancreas epithelial cells [82]. However, the intersection between TGFβ signaling and the redox system is complex, and involves several other effectors. For example, PDAC cells with a stable knockdown of TGFβ ligands show increased NOX4-dependent ROS production, and activation of several stress-activated protein kinases (SAPKs) including p38 and JNK. These cells also demonstrated a diminished expression of TRX and GSTM1, which inhibit the actions of ASK1. The authors concluded that, in the context of TGFβ-deficiency, ASK1 was activated and induced cell death via p38/JNK activation and/or the induction of ER stress [83]. Again, these data suggest that the interactions between TGFβ and redox signaling are highly complex, and warrant further study.

Beyond alterations to redox signaling, TGFβ-induced EMT in PDAC also involves several other target genes that have not classically been associated with TGFβ signaling. One such example is the gene Menin, which coordinates interactions between TGFβ signals and C/EBPβ to balance growth inhibition and EMT [84]. Specifically, Menin overexpression decreased the expression of C/EBPβ and increased TGFβ-induced EMT through alterations to histone acetylation [84]. TGFβ-induced EMT also seemingly requires BCL9L, as cells with a loss of BCL9L retain a strong epithelial phenotype irrespective of prolonged incubation with TGFβ [85]. Additionally, through both SMAD-dependent and SMAD-independent pathways, TGFβ induces the expression of the co-stimulatory protein B7-1, which is required for TGFβ-induced EMT as well as PDAC cell migration and invasion [86]. Recent evidence also suggests that the inflammation-associated protein leucine-rich alpha-2 glycoprotein (LRG) potentiates TGFβ-induced EMT in PDAC cells, though the precise mechanism through which LRG enhances TGFβ signaling remains unclear [87].

TGFβ-induced EMT also involves crosstalk with additional signaling pathways, including the Hippo-YAP pathway. PDAC cells with a loss of YAP1 are poorly sensitive to TGFβ-induced EMT, and TGFβ treatment appears to preferentially stabilize the YAP1-2 splice variant and enhance its nuclear localization in an AKT-dependent manner [88]. TGFβ signaling in PDAC also involves crosstalk with RAC1 and its related isoform RAC1b, which may have important roles in the TGFβ-induced EMT [89]. Further, TGFβ-mediated downregulation of PTEN also appears to involve NFκB. Consistent with observations that TGFβ downregulates PTEN in SMAD4-null PDAC lines, TGFβ induced IκBα phosphorylation, thereby leading to the increased activation of NFκB, and subsequent transcriptional repression of PTEN. Inhibition of IκBα led to the de-repression of PTEN, as well as reduced TGFβ-induced cell migration. This was reversed upon restoration of SMAD4, but not knockdown of SMAD2 and/or SMAD3 [90]. Recent evidence also appears to implicate PLEXIND1 in pathologic TGFβ signaling, which acts as a co-receptor to promote tumor growth and reduce E-cadherin expressing in tumor cells with oncogenic KRAS mutation. However, these results were not observed in cells with wild-type KRAS, in which PLEXIND1 functioned as a tumor suppressor [91]. Combined, these observations underscore both the high degree of complexity relating to non-SMAD TGFβ signaling, as well as the many intersections between SMAD and non-SMAD arms of the TGFβ pathway.

Finally, TGFβ signaling involves a variety of microRNAs (miRNAs) with diverse and often poorly defined roles in tumor cell biology [92]. For example, miR-10b expression correlates with disease aggressiveness in PDAC, markedly enhances the effects of TGFβ on EMT and cell migration, and facilitates oncogenic crosstalk between TGFβ and EGF signaling pathways [93]. Additionally, SMAD-dependent TGFβ signals upregulate the MIR100HG long non-coding RNA (lncRNA), which contains the oncogenic miRNAs miR-100 and miR-125b, as well as the tumor suppressive let-7a miRNA precursor. While this corresponded to an increased expression of miR-100 and miR-125b, the authors determined that levels of let-7a were unchanged due to the TGFβ-induced upregulation of LIN28B, thereby blocking the maturation of let-7a. Inhibition of miR-100 or miR-125b diminished cellular responses to TGFβ, and interfered with signaling pathways related to both p53 and cell–cell junctions [94]. Other miRNAs also appear to have a role in negatively regulating TGFβ signaling. For example, miR-141 mimics inhibited the activation of TGFβ signals in PDAC cells [95], and miR-145 suppresses EMT by inhibiting TGFβ signaling [96]. miR-107 also appears to promote PDAC cell proliferation, invasion, and migration by targeting type 3 TGFβ receptor (TGFBR3) [97]. However, as TGFBR3 is not thought to contribute to classical TGFβ signaling and instead predominantly acts as a ligand trap [98,99], the impact of these findings on the TGFβ pathway are unclear and warrants additional study.

## 4. TGFβ in Fibrosis and Stromal Cell Biology

In addition to its effects in tumor cells, TGFβ has several roles within the tumor microenvironment [5]. PDAC is associated with a dense, desmoplastic tumor stroma predominantly comprised of extracellular matrix (ECM) proteins, mesenchymal cells, and leukocytes [100,101,102,103]. The tumor stroma has been found to promote disease progression, metastasis, and therapeutic resistance through a number of mechanisms. These include the mechanical induction of intracellular signaling that promote pancreatic carcinogenesis [104], as well as paracrine signaling events directing a variety of tumor cell processes [105]. TGFβ is known to regulate the heterogeneous populations of mesenchymal cells residing in the tumor stroma, many of which are critical to the incidence and progression of PDAC [106,107,108].

For example, a recent study utilized high-throughput proteomics to characterize ECM proteins in the normal pancreas, PanIN lesions, as well as human and murine PDAC specimens. The authors identified an early upregulated group of matrisome proteins in PanIN lesions that are further upregulated in PDAC tumors. They also found that stromal cells produce over 90% of the ECM mass, with the remaining 10% is attributed to the tumor cells themselves, and in both cell types TGFβ1 was upstream of more matrisome proteins than any other gene evaluated [109]. Additionally, TGFβ mRNA strongly correlates with that of several collagen family members in PDAC specimens [110], and multiple studies have linked TGFβ signaling to MMP-mediated ECM remodeling [111,112]. Accordingly, transgenic mouse models of cystic papillary neoplastic lesions display a significant reduction in collagen expression when crossed to mice with a heterozygous deletion of *Tgfbr1* [17].

Additional studies have explored the means through which TGFβ signaling enhances PDAC-associated fibrosis, with most focusing on pancreatic stellate cells (PSCs) and cancer-associated fibroblasts (CAFs). PSCs are a population of myofibroblast-like cells, and are considered the major cellular component of PDAC stroma [113] and one of the main sources of collagen within the PDAC TME [106]. Contrasting its growth inhibitory effects in well-differentiated epithelial cells, TGFβ appears to promote the activation and proliferation of PSCs, as well as enhance PSC migration and the deposition of ECM proteins through both SMAD and non-SMAD signaling pathways [114,115,116,117,118]. Notably, exogenous TGFβ enhances de novo collagen formation in cultured PSCs, both at the mRNA and protein levels, underscoring the pro-fibrotic role for TGFβ in PDAC [17,115,119].

In addition to being highly TGFβ-responsive, PSCs are a primary source of TGFβ ligands. PSCs secrete high concentrations of TGFβ [120], which exceed that of PDAC cells in vitro [17]. Furthermore, TGFβ promotes its own expression in PSCs through a positive feedback mechanism [17]. This PSC-derived TGFβ has several effects on nearby PDAC cells, leading to hyperactive responses to exogenous TGFβ1 and enhancing EMT and stemness in part through the repression of L1 cell adhesion molecules (L1CAM) [17,121].

As mentioned, TGFβ is also a central regulator of cancer-associated fibroblasts (CAFs) in PDAC. CAFs are a heterogeneous population of mesenchymal cells and can play both tumor-enhancing and tumor-suppressive roles in pancreatic carcinogenesis [122]. For example, the depletion of CAFs accelerates PDAC formation in vivo, leading to local immune suppression, poor tumor differentiation, and poor survival [123,124,125]. However, CAFs also enhance tumor cell proliferation by providing metabolic support through amino acids including alanine, which supports lipid and amino acid biosynthesis [126,127]. CAFs are also the source of several tumor-enhancing cytokines, growth factors, and other immunomodulators, all of which can serve to enhance tumor formation [128,129,130,131].

**Figure 2:**
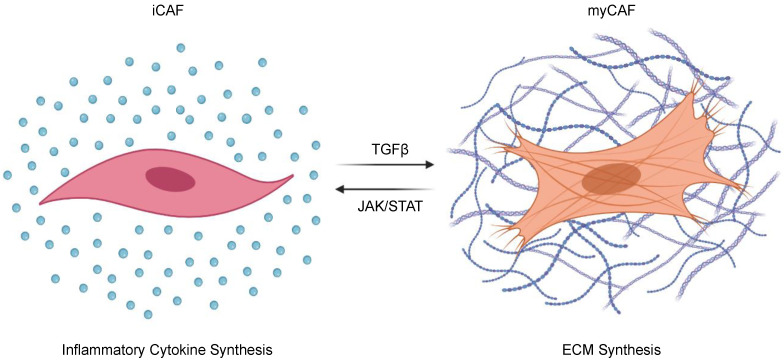
The effects of TGFβ signals on cancer-associated fibroblast polarization. Cancer-associated-fibroblasts (CAFs) can be sub-categorized into inflammatory CAFs (iCAFs) that enhance local inflammatory cues through the secretion of cytokines such as interleukin 6 (IL-6) and leukemia inhibitory factor (LIF); and myofibroblastic CAFs (myCAFs) that hyper-secrete extracellular matrix (ECM) proteins. The balance between iCAFs and myCAFs is determined by competition between TGFβ and JAK/STAT signaling pathways, where TGFβ signaling polarizes CAFs toward a pro-fibrotic myCAF phenotype.

Recently, TGFβ has been demonstrated to regulate the pro- and anti-tumorigenic properties of CAFs through phenotypic change (Figure 2). CAFs are both plastic and heterogeneous [132], and can be sub-categorized into inflammatory CAFs (iCAFs) that enhance local inflammatory cues through the secretion of cytokines such as interleukin 6 (IL-6) and leukemia inhibitory factor (LIF), and myofibroblastic CAFs (myCAFs) that express α smooth muscle actin (αSMA) and contribute to ECM deposition [133,134,135]. The balance between iCAFs and myCAFs is determined by competition between TGFβ and JAK/STAT signaling pathways. When TGFβ signals are inhibited, JAK/STAT signaling and pro-tumoral iCAFs dominate. Conversely, in the absence of JAK/STAT signals, TGFβ will dominate and shift CAFs toward a myCAF phenotype, increasing ECM deposition and restraining tumor progression in vivo [134]. In addition to increasing ECM deposition, myCAFs have important immunomodulatory effects, namely the subset expressing Leucine-Rich Repeat Containing 15 (LRRC15). These LRRC15-expressing myCAF-like cells contribute to the failure of immune checkpoint inhibition in PDAC, which is consistent with previous observations suggesting that TGFβ-induced ECM genes link CAFs to immune evasion and the failure of cancer immunotherapy [136,137]. A recent study has identified another subset of poorly immunogenic CAFs that express CD105, an auxiliary receptor to the TGFβ signaling complex. These CD105-expressing CAFs were more abundant than their CD105-non-expressing counterparts, were transcriptionally more responsive to TGFβ signaling, and demonstrated facilitated tumor growth in vivo. As these phenotypic differences appeared to be independent of myCAF and iCAF markers, CAF heterogeneity warrants additional study, as do the contributions of TGFβ signals to mesenchymal cell biology in PDAC [138].

## 5. TGFβ and the Immune Microenvironment

**Figure 3:**
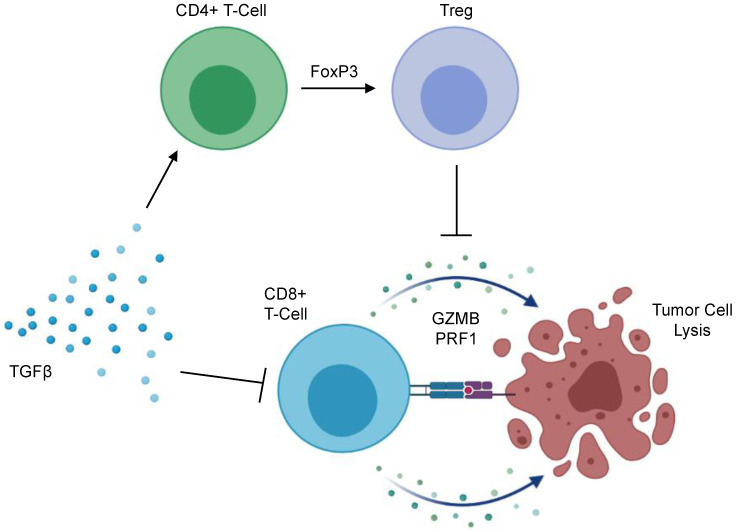
Mechanisms of TGFβ-mediated immunosuppression in the PDAC tumor microenvironment. TGFβ signaling impedes the anti-tumor immune surveillance program through several mechanisms. TGFβ ligands have a pronounced inhibitory effect on the genesis and effector function of CD8^+^ cytotoxic T-cells, leading to the transcriptional repression of functional cytokines including Granzyme B (GZMB), Perforin 1 (PRF1), and interferon-γ. As a result, these CD8^+^ T-cells remain refractory from full activation and fail to mount a full anti-tumor immune response. Additionally, TGFβ signaling acts on CD4^+^ helper T-cells, acting to upregulate Forkhead box protein P3 (FoxP3). This leads to the peripheral conversion of CD4^+^ helper T-cells to immunosuppressive regulatory T-cells (Tregs). Tregs suppress sterilizing immunity via the secretion of suppressive cytokines such as TGFβ and IL-10, as well through the surface expression of immune checkpoint molecules.

The immunosuppressive effects of TGFβ signaling are well documented [139] with early reports demonstrating that TGFβ signals have a pronounced inhibitory effect on the genesis and effector function of cytotoxic T-lymphocytes (CTLs) [140]. This has been affirmed through subsequent mechanistic studies indicating that TGFβ impedes the effector function of CTLs through the canonical SMAD pathway, leading to the transcriptional repression of functional cytokines including interferon-γ and granzyme B [141], the latter being an anti-tumor serine protease found in CTL-associated cytotoxic granules with important roles in anti-tumor immunity [142,143]. Accordingly, TGFβ is emerging as a key mediator of immune evasion in several cancers—including PDAC (Figure 3). Several in vivo studies have demonstrated that CTLs deficient in TGFβ signals are capable of mounting a robust anti-tumor immune response [144,145,146,147]. Accordingly, PDAC tumors with a higher expression of TGFβ display reduced levels of granzyme B, and the adoptive transfer of CTLs deficient in TGFBR1 led to the T-cell mediated regression of early-stage pancreatic intraepithelial neoplasms (PanINs) in mice [17].

These observations have led to extensive research into TGFβ signal inhibition as a means of reactivating the anti-tumor immune surveillance program. Select studies have explored the single agent efficacy of TGFβ signal inhibition in mouse models of advanced PDAC; however, such approaches fail to significantly evoke a functional anti-tumor immune response or enhance survival [148,149]. As murine PDAC tumors with a loss of TGFβ signaling display increased expression of the clinically actionable immune checkpoint PD-L1 [148,149,150], subsequent studies have evaluated the combined inhibition of TGFβ signaling and either PD-L1 or its receptor PD-1. This approach has shown early promise, as transgenic models of PDAC with either the genetic [149] or pharmacologic [148] inhibition of TGFBR1 display increased sensitivity to PD-1 inhibition, with similar results observed in both a subcutaneous xenograft model and orthotopic tumor models using pH-responsive clustered nanoparticles to inhibit both TGFβ and PD-L1 [151]. Importantly, *Pdx1-Cre × LSL-Kras^G12D^ × Tp53^R172H^* (KPC) mice administered a combination of the TGFBR1 inhibitor Galunisertib and an anti-PD-1 antibody showed improved overall survival, as well as a substantial increase in tumor-infiltrating lymphocytes, granzyme B deposition, and apoptosis in remaining areas of neoplastic disease [148].

Though encouraging, it is important to note that responses to combined TGFβ and PD-1 inhibition were not uniform in this study. Though nearly all mice in the dual treatment arm had a survival advantage, survival duration was extremely varied, and the majority of mice still succumbed to their disease within the 180-day treatment interval [148]. In a subsequent study, long-term administration of the anti-neoplastic agent Gemcitabine markedly enhanced responses to concomitant TGFβ and PD-1, leading to more durable and uniform immune responses in KPC mice [149]. This was presumed due to the enhanced antigen presentation induced by Gemcitabine, as well as increased levels of TGFβ within the tumor microenvironment [149]. Hence, this and similar combination strategies warrant continued exploration in the treatment of PDAC. However, it is important to note that the success of dual TGFβ and PD-L1/PD-1 inhibition appear to be highly dependent on the model system used [152]. Hence, the use of multiple, complementary model systems is recommended, including new tools for immunology research including ex vivo slice cultures, patient-derived xenografts in partially humanized mice, and large animal models of PDAC [153,154,155,156,157].

Beyond its effects on CD8^+^ T-cells in PDAC, TGFβ also directs CD4^+^ T-cell function, particularly regarding peripheral regulatory T-cell (Treg) conversion. Tregs are a unique subset of CD4^+^ T-cells, predominantly defined by the expression of the transcription factor Forkhead box protein P3 (FoxP3) [158]. Contrasting helper CD4^+^ T-cells, Tregs maintain immune homeostasis and self-tolerance by suppressing the activity of other immune cell subsets [159,160,161,162]. Tregs are frequent in most human cancers, and largely converted within the TME [163]. In PDAC, increased tumor-infiltrating Tregs predicts for reduced CD8^+^ T-cell infiltration, as well as a poor prognosis [164]. In vivo, Tregs negatively regulate tumor-associated dendritic cells, limiting their expression of the costimulatory ligands necessary for CD8^+^ T-cell activation. Additionally, Treg ablation evokes an effective anti-tumor immune response in implanted murine PDAC tumors [165]. However, though often considered tumor permissive, the deletion of Tregs accelerates tumor formation in transgenic models of murine PDAC, associated with a loss of myCAFs within the TME, as well as an increase in immunosuppressive myeloid cells and pathological CD4^+^ T cell responses [166]. Hence, the contribution of Tregs to PDAC pathobiology warrants continued exploration. The role of TGFβ signals should also be considered, particularly in light of observations that TGFβ inhibition can deplete Tregs and enhance immune responses in tumor-bearing mice administered a GM-CSF secreting allogeneic pancreas tumor vaccine (GVAX) [167], and that the combination of anti-CD25-mediated Treg depletion and TGFβ inhibition potentiates the effects of anti-PD-1 in vivo [168].

TGFβ signaling also has important effects on additional immune cells in PDAC, though these are largely unclear. While tumor-bearing mice with a systemic administration of TGFβ showed no difference with respect to myeloid-derived suppressor cells (MDSCs) or dendritic cells (DCs), both cell types were reduced in liver metastases. Although TGFβ did not affect the number of tumor-infiltrating macrophages (TAMs) in primary or metastatic tumors, TGFβ treatment enhanced the percent of TAMs positive for PD-L1 [169]. TGFβ signaling has also been implicated in the tumor-enhancing properties of macrophages in PDAC, as TGFβ signal inhibition abolished the macrophage-induced EMT in tumor cells [170], consistent with observations that macrophage-derived exosomal microRNA-501-3p enhances PDAC tumorigenesis via the suppression of TGFBR3, thereby leading to activating the TGFβ pathway [171]. Finally, TGFβ signaling also appears to inhibit the actions of natural killer (NK) cells in PDAC. Tumor-derived extracellular vesicles contain TGFβ ligands, which suppress NK cell activation via the SMAD pathway, reducing the expression of NKG2D, CD107a, TNFα, and INFγ [172]. This is supported by additional evidence suggesting that TGFβ impairs the NK-mediated lysis of PDAC cells in vitro [173]. Given the role of these and other cell types in PDAC pathobiology as well as therapeutic responses to immune checkpoint inhibition, the effects of TGFβ in these cells warrant continued exploration.

## 6. Clinical Trials Exploring TGFβ Signal Inhibition in PDAC

Given the established roles of TGFβ signaling in PDAC pathobiology, several TGFβ pathway inhibitors are emerging in clinical trial [174], often in combination with chemo-, immuno-, or radiation-therapy (Table 1). One such example used AP 12009 (trabedersen), a phosphonothioate antisense oligodeoxynucleotide targeting the TGFβ2 transcript that has previously shown preclinical efficacy in PDAC [175]. Early results from 37 patients with metastatic PDAC have been posted. AP 12009 was well tolerated, with no maximum tolerated dose reached. Additionally, AP 12009 extended overall survival to 14.7 months when followed by chemotherapy, however, the significant benefit was reduced when AP 12009 was administered after chemotherapy [176].

The TGFBR1-inhibitor Galunisertib was also explored in PDAC patients, as a monotherapy and in combination with Gemcitabine. In a recent phase Ib, 14 patients with metastatic PDAC were administered between 80 and 300 mg Galunisertib twice daily alone or in combination with standard dose Gemcitabine. Of the 13 evaluable patients, 5/13 showed stable disease, 1/13 had a partial response, and 6/13 had progressive disease [177]. As no dose-limiting toxicities were observed, the phase II portion of this trial utilized the 300 mg dose and included 156 patients with non-resectable, advanced, or metastatic PDAC. Patients were given either standard dosing Gemcitabine, or Gemcitabine and Galunisertib as described. The median overall survival was 7.1 months for the Gemcitabine group, and 8.9 months for the group receiving Galunisertib and Gemcitabine. The addition of Galunisertib similarly extended progression-free survival from 2.86 to 4.11 months. Galunisertib did not significantly increase the rate of serious adverse events, which were reported in 54% of patients in the combination arm, and 50% of those in the control group. Common adverse effects included anemia, neutropenia, thrombocytopenia, vomiting, constipation, peripheral edema, fever, and fatigue [177].

Given the immunomodulatory role of TGFβ signals, Galunisertib is also being evaluated in combination with immune checkpoint inhibition. A recent phase Ib trial evaluated Galunisertib in combination with the anti-PD-L1 antibody Durvalumab in 37 patients recurrent/refractory metastatic PDAC previously treated with ≤2 systemic regimens. Consistently with previous reports, Galunisertib was well tolerated, and no dose-limiting toxicities were observed at the highest dose of 150 mg. This dose was selected for the phase II component, which included 32 patients meeting the above criteria. Here, 1/32 demonstrated a partial response, 7/32 showed stable disease, and 15/32 had progressive disease. The disease control rate was 25.0%, with a median overall survival of 5.72 months and median progression-free survival of 1.87 months. Five patients experienced a grade 3/4 treatment-related adverse event in the form of elevated AST/ALT, neutropenia, anemia, and/or lymphopenia. Though encouraging given the highly advanced disease in this cohort, the authors recommended the continued investigation of this combination as an earlier line of treatment or in combination with predictive biomarkers for TGFβ inhibition [178].

A recent phase I trial in several solid cancers explored the utility of M7824 (bintrafusp alfa), a bifunctional fusion protein composed of a monoclonal antibody against PD-L1 fused to a TGFβ ligand trap. This study included 19 heavily pretreated cancer patients, four of which developed grade ≤ 3 in the form of skin infection secondary to localized bullous pemphigoid, increased lipase levels without pancreatitis, colitis with associated anemia, and gastroparesis with hypokalemia. Efficacy was seen across all treatment groups, and the maximum tolerated dose was not reached. This study included five PDAC patients, and only one patient with locally advanced PDAC deficient in DNA mismatch repair (dMMR) with high microsatellite instability (MSI-H) showed a partial response at a dose of 3 mg/kg. This patient had a durable response that persisted until disease progression after 10.5 months [179]. M7824 was also evaluated in combination with Gemcitabine in a recent clinical phase Ib/II trial enrolling a small number of patients with heavily pre-treated PDAC. All patients in the study experienced grade 3/4 adverse events, with 66% developing anemia, 33% developing thrombocytopenia, and 16% developing upper GI hemorrhage, pleural effusion, or thromboembolism, and the study was terminated after a patient died from ICI-induced hepatitis (NCT03451773).

Additional clinical trials are ongoing (Table 2), including the combination of M7824 in combination with stereotactic body radiation therapy (SBRT) and the immunocytokine M9241, which is composed of two IL-12 heterodimers fused to an antibody with affinity for both single-strand and double-strand DNA as a neoadjuvant treatment (NCT04327986). Another phase Ib/II trial is evaluating the combination of SHR-17011, a bifunctional fusion protein targeting PD-L1 and TGFBR2, and Gemcitabine/Albumin-Paclitaxel as first-line therapy in patients with advanced or metastatic PDAC (NCT04624217). Additional studies are also evaluating the safety and efficacy of Vactosertib (TEW-7197), a small molecule inhibitor that blocks intracellular signaling by TGFβ signals via the inhibition of the TGFBR1 family member activin receptor-like kinase 5 (ALK5). One such example is a phase Ib trial utilizing Vactosertib in combination with folinic acid, 5-Fluorouracil (5-FU), and Oxaliplatin (FOLOX) in patients with metastatic PDAC who previously progressed on Gemcitabine/Nab-Paclitaxel (NCT03666832), as is the combination of Vactosertib with liposomal Irinotecan and 5-FU (NCT03666832).

## 7. Conclusions

TGFβ signaling has several important and often contradictory roles within the pancreatic TME. Although TGFβ signals can exert potent tumor-suppressive effects through SMAD-mediated cell cycle arrest, TGFβ also accelerates pancreatic tumorigenesis by enhancing EMT, fibrosis, and immune evasion. Although TGFβ has a clearly dual function in tumor prevention and carcinogenesis, approximately half of PDAC patients demonstrate a loss of the TGFβ effector SMAD4. In addition to carrying a particularly poor prognosis, SMAD4-deleted PDAC patients are presumed to be insensitive to the growth inhibitory effects of TGFβ signaling, and yet may retain the more detrimental effects of TGFβ signals in the TME. As TGFβ inhibitors show early promise in the treatment of PDAC patients, the effects of TGFβ signals on both epithelial and non-epithelial cell types warrant continued exploration in the hope of both identifying the most effective combination strategies including TGFβ inhibitors, as well as the patients in which TGFβ inhibition will be most effective.

## Figures and Tables

**Table 1:** Results of select clinical trials exploring TGFβ inhibitors in PDAC patients.

TGFβ Inhibitor	Additional Therapy	Phase	Number of Patients	Prior Lines of Therapy	Response Rate	Median PFS	Median OS	Ref
Galunisertib	Durvalumab	Ib	37	≤2	3.1%	1.87	5.72	[178]
Galunisertib	-	II	52	≥1	3.8%	2.86	7.10	[177]
Galunisertib	Gemcitabine	II	104	≥1	10.6%	4.11	8.90	[177]
M7824	Gemcitabine	II	7	≥1	NR	1.40	3.50	NCT 03451773
AP 12009	-	Ib	62	-	NR	NR	NR	NCT 00844064
Galunisertib	Gemcitabine	Ib	6	-	NR	NR	NR	NCT 02154646

NR = not reported.

**Table 2:** Ongoing clinical trials exploring TGFβ inhibitors in PDAC patients that have yet to post results.

TGFβ Inhibitor	Additional Therapy	Phase	Enrolment Criteria	Prior Treatments	Clinical Trial Number
SHR-1701	Gemcitabine /Nab-Paclitaxel	Ib/II	Advanced or Metastatic PDAC	-	NCT04624217
Vactosertib (TEW-7197)	FOLFOX	Ib	Metastatic PDAC	Gemcitbine /Nab-Paclitaxel	NCT03666832
Vactosertib (TEW-7197)	Nal-Irinotecan /5-FU	Ib	Metastatic PDAC	Gemcitabine /Nab-Paclitaxel	NCT04258072
M7824	M9241/RT	I/II	Advanced or Metastatic PDAC	-	NCT04327986

RT = radiotherapy.

## Abbreviations

Pancreatic ductal adenocarcinoma	(PDAC)
Transforming Growth Factor β	(TGFβ)
Tumor microenvironment	(TME)
Epithelial-to-mesenchymal transition	(EMT)
Pancreatic stellate cells	(PSCs)
Cancer-associated fibroblasts	(CAFs)
Extracellular matrix	(ECM)
MicroRNAs	(miRNAs)
DNA mismatch repair	(dMMR)
High microsatellite instability	(MSI-H)
